# Comparison of the oxidative phosphorylation (OXPHOS) nuclear genes in the genomes of *Drosophila melanogaster*, *Drosophila pseudoobscura *and *Anopheles gambiae*

**DOI:** 10.1186/gb-2005-6-2-r11

**Published:** 2005-01-31

**Authors:** Gaetano Tripoli, Domenica D'Elia, Paolo Barsanti, Corrado Caggese

**Affiliations:** 1 University of Bari, DAPEG Section of Genetics, via Amendola 165/A, 70126 Bari, Italy; 2 CNR, Institute of Biomedical Technology, Section of Bari, via Amendola 122/D, 70126 Bari, Italy

## Abstract

**Background:**

In eukaryotic cells, oxidative phosphorylation (OXPHOS) uses the products of both nuclear and mitochondrial genes to generate cellular ATP. Interspecies comparative analysis of these genes, which appear to be under strong functional constraints, may shed light on the evolutionary mechanisms that act on a set of genes correlated by function and subcellular localization of their products.

**Results:**

We have identified and annotated the *Drosophila melanogaster*, *D. pseudoobscura *and *Anopheles gambiae *orthologs of 78 nuclear genes encoding mitochondrial proteins involved in oxidative phosphorylation by a comparative analysis of their genomic sequences and organization. We have also identified 47 genes in these three dipteran species each of which shares significant sequence homology with one of the above-mentioned OXPHOS orthologs, and which are likely to have originated by duplication during evolution. Gene structure and intron length are essentially conserved in the three species, although gain or loss of introns is common in *A. gambiae*. In most tissues of *D. melanogaster *and *A. gambiae *the expression level of the duplicate gene is much lower than that of the original gene, and in *D. melanogaster *at least, its expression is almost always strongly testis-biased, in contrast to the soma-biased expression of the parent gene.

**Conclusions:**

Quickly achieving an expression pattern different from the parent genes may be required for new OXPHOS gene duplicates to be maintained in the genome. This may be a general evolutionary mechanism for originating phenotypic changes that could lead to species differentiation.

## Abstract 2

An analysis of nuclear-encoded oxidative phosphorylation genes in *Drosophila* and *Anopheles* reveals that pairs of duplicated genes have strikingly different expression patterns.

## Background

The accessibility of whole-genome sequence data for several organisms, together with the development of efficient computer-based search tools, has revolutionized modern biology, allowing in-depth comparative analysis of genomes [1-4]. In many cases, comparisons among species at various levels of divergence have helped to define protein-coding genes, recognize nonfunctional genes, and find regulatory sequences and other functional elements in the genome. When applied to a set of genes correlated by function and/or subcellular localization of their products, intra- and interspecies comparative analyses can be especially efficient tools to obtain information on the functional constraints acting on the evolution of the gene set and on the mechanisms regulating its coordinate expression.

A set of genes present in all eukaryotic genomes and expected to be subject to peculiar evolutionary constraints is represented by the genes involved in oxidative phosphorylation (OXPHOS), the primary energy-producing process in all aerobic organisms [5]. To generate cellular ATP, OXPHOS uses the products of both nuclear and mitochondrial genes, organized in five large complexes embedded in the lipid bilayer of the inner mitochondrial membrane. Except for complex II, which is formed by four proteins encoded by nuclear genes, the other respiratory complexes depend on both mitochondrial and nuclear genomes; so, assembling the OXPHOS complexes and fine tuning their activity to satisfy cell- and tissue-specific energy demands requires specialized regulatory mechanisms and evolutionary strategies to optimize the cross-talk between the two genomes and ensure the coordinated expression of their relevant products.

Analysis of co-regulated mitochondrial and nuclear genes, and of the transcription factors regulating the functional network they constitute, might also be a useful approach to investigate the origin of mitochondrial dysfunction in humans. Disorders of mitochondrial oxidative phosphorylation are now recognized as the most common inborn errors of metabolism, affecting at least one in 5,000 newborn children [6]. In this context, the expanding spectrum of identified mitochondrial proteins provides an opportunity to test a whole new range of candidate genes whose mutations may be responsible for common human diseases. For example, a recent study by Mootha *et al*. [7] suggests a promising strategy for clarifying the molecular etiology of mitochondrial pathologies by profiling the tissue-specific expression pattern of candidate mitochondrial proteins.

Despite the long evolutionary divergence time, many key pathways that control development and physiology are conserved between *Drosophila *and humans, and about 70% of the genes associated with human disease have direct counterparts in the *Drosophila *genome [8,9]. For example, the potential role of *Drosophila *as a model system for understanding the molecular mechanisms involved in human genetic disease is validated by the recent identification of a *Drosophila *mutation causing a necrotic phenotype that mimics in detail the diseases that arise from serpin mutations in humans [10].

It has been suggested that comparisons between *D. melanogaster *and other species of the genus *Drosophila *could provide a model system for developing and testing new algorithms and strategies for the functional annotation of complex genomes [3]. To obtain new information on the evolution of a set of genes that control a basic biological function by encoding products targeted to a specific cellular compartment, we have performed a comparative analysis of the OXPHOS genes of *D. melanogaster *and *D. pseudoobscura*; the complete genome of the latter was recently made available by the Baylor Human Genome Sequencing Center. These two species are the only species of the *Drosophila *genus for which whole-genome sequence data exist at present [11-13]. We also took advantage of the complete sequence of the *A. gambiae *genome [14] to compare the *Drosophila *OXPHOS genes with those of this more distantly related dipteran (the divergence time between *D. melanogaster *and *A. gambiae *is thought to be approximately 250 million years, as compared to 46 million years between *D. melanogaster *and *D. pseudoobscura *[15,16]). Although extensive reshuffling within and between chromosomal regions is known to have occurred since the divergence of *Anopheles *from *Drosophila *[4,17,18], we show that in these organisms the conservation of the OXPHOS genes is still sufficient to permit their meaningful comparison.

Here we report the identification of 78 *D. pseudoobscura *and 78 *A. gambiae *genes representing the counterparts of *D. melanogaster *OXPHOS genes which, in turn, were previously identified as putative orthologs of human OXPHOS genes [19]. We have annotated these genes, taking into account conservation in amino-acid sequence, intron-exon structure, intron length, and the presence of duplications in the genome. The conservation of genomic organization and evidence from evolutionary trees based on sequence similarity suggest that these genes are one-to-one orthologs in the three species, and that in many cases they originated (produced?) duplicates by transpositional and/or recombinational events during evolution. We have identified in the three dipteran genomes a total of 47 genes that probably originated by duplication of the above-mentioned genes, and we show that the duplicate gene has usually acquired a pattern of expression strikingly different from that of the gene from which it derived. Moreover, when the comparison is possible, the gene duplicate almost always shows a strongly testis-biased expression, in contrast to the soma-biased expression of its parent gene.

## Results and discussion

### Identification and comparative annotation of *D. pseudoobscura *and *A. gambiae *OXPHOS genes

We have previously reported [19] the identification of 285 *D. melanogaster *nuclear genes encoding mitochondrial proteins that represent the counterparts of human peptides annotated in the Swiss-Prot database as mitochondrial [20]. On the basis of comparative evidence obtained by BLASTP analysis, 78 of these genes are involved in the OXPHOS system, encoding 66 proteins known to be components of the five large respiratory complexes and 12 proteins involved in oxidative phosphorylation as accessory proteins. To identify the putative counterparts of the *D. melanogaster *OXPHOS genes in *D. pseudoobscura *and *A. gambiae *we performed a TBLASTN search [13,21] on the whole genome sequences of these species using the amino-acid sequences of the 78 *D. melanogaster *peptides as queries. Sequences giving the best reciprocal BLAST hits were tentatively assumed to identify functional counterparts in two species if they could be aligned over at least 60% of the gene length and the BLAST E-score was less than 10^-30^. By these criteria, all the 78 *D. melanogaster *OXPHOS genes investigated have a counterpart both in *D. pseudoobscura *and in *A. gambiae*. To better compare the structure of the OXPHOS genes in the three dipteran species, we used the predicted coding sequences as queries for a search of expressed sequence tags (EST) [21], and used the retrieved sequences to annotate the transcribed noncoding sequences of the *A. gambiae *genes investigated. Although little EST information is available for *D. pseudoobscura*, it was still possible to predict unambiguously the exon-intron gene structure of the OXPHOS genes in this species, as well as the amino-acid sequence of their full-length products, by exploiting the high level of similarity with *D. melanogaster*. The results of BLAST analysis, together with the construction of phylogenetic trees that also include other genes that show lesser but still significant sequence similarity to the 78 genes assumed to be one-to-one orthologs in the three species investigated (see below), strongly suggest that the newly identified *D. pseudoobscura *and *A. gambiae *genes are the functional counterparts of the 78 *D. melanogaster *genes used as probes.

Table 1 lists the 78 putative orthologous OXPHOS genes in the three dipteran genomes and their cytological location. For each gene, a record showing the gene map and reporting the annotated genomic sequences as well as the mRNA and protein sequences is available and can be queried at the MitoComp website [22] (see also Additional data files). MitoComp also compares the structure of the *D. melanogaster*, *D. pseudoobscura *and *A. gambiae *putative orthologous genes and their duplications when present (see below), and aligns the orthologous coding sequences (CDS), and also aligns their deduced amino-acid products with the corresponding human protein.

### Amino-acid sequence comparison

**Figure 1:**
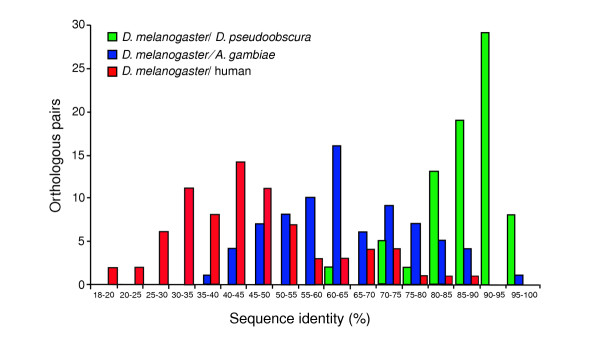
Histogram of pairwise sequence identities between the unprocessed products of 78 orthologous *D. melanogaster*, *D. pseudoobscura*, *A. gambiae *and human OXPHOS genes.

For the products of the OXPHOS genes investigated, the *D. melanogaster*/*D. pseudoobscura *average amino-acid sequence identity is 88%, compared to 64% between *D. melanogaster *and *A. gambiae*. Figure 1 shows the frequency distribution of sequence identities, and Additional data file 1 lists all pairwise identity values between the products of the 78 OXPHOS genes when orthologous *D. melanogaster*/*D. pseudoobscura*, *D. melanogaster*/*A. gambiae *and *D. melanogaster*/human gene products are compared. A multiple alignment of each cluster of homologous proteins is shown at the MitoComp website [22].

It should be kept in mind that identity values reported in Figure 1 and in the table in Additional data file 1 were calculated on the whole sequence of the predicted unprocessed proteins; they are much higher if the putative amino-terminal pre-sequences are excluded, since such sequences, possessed by most mitochondrion-targeted products, show little amino-acid sequence conservation [23,24], although they do share specific physicochemical properties [25,26]. When only the predicted mature protein is considered, the average percentage identity increases to 90% between *D. melanogaster *and *D. pseudoobscura*, and to 70% between *D. melanogaster *and *A. gambiae*.

A striking example of evolutionary conservation is provided by the genes encoding cytochrome *c *(an essential and ubiquitous protein found in all organisms) in the three dipteran species: the amino-acid sequences of the gene products are identical in *D. melanogaster *and *D. pseudoobscura*, whereas 96% identity is preserved between *Drosophila *and *Anopheles*. Coding sequences are also extremely conserved, suggesting that the nucleotide sequence itself is subject to strong evolutionary constraints, maybe due to codon usage bias. Only synonymous substitutions (21 out of 108 codons) were found on comparing *D. melanogaster *and *D. pseudoobscura *cytochrome *c *coding sequences, whereas 28 synonymous substitutions and only four nonsynonymous substitutions were observed between *D. melanogaster *and *A. gambiae *(see MitoComp website [22]).

### Gene structure comparisons

It is well known that a given function may be supplied in different species by genes that are not directly derived from a common ancestor, that is, by paralogous, not orthologous, genes. Therefore, we thought it would be interesting to compare the structural organization of the OXPHOS genes in the three species investigated, on the principle that it should be possible to infer derivation from a common ancestor, that is, 'structural orthology', if an identical or very similar overall structure was preserved. As the introns of the putative orthologous OXPHOS genes in the three species are, as expected, too divergent in DNA sequence to be aligned, we used conservation of number of introns, conservation of their location in the coding sequence, and preservation of the reading frame with respect to the flanking exons as our primary criteria.

**Figure 2:**
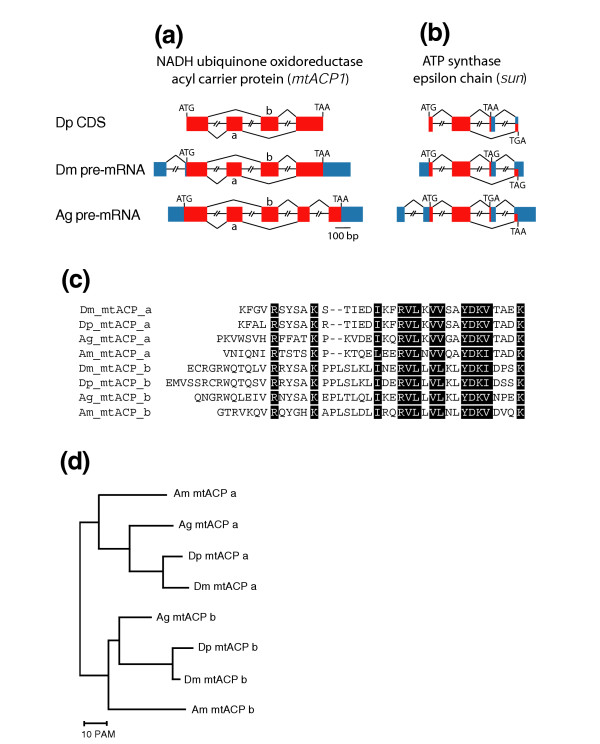
Conservation of alternative splice variants of two OXPHOS genes in *D. melanogaster*, *D. pseudoobscura *and *A. gambiae*. **(a,b) **Schematic representation and comparison of intron-exon structure of the genes encoding the NADH ubiquinone-oxidoreductase acyl carrier protein and the ATP synthase epsilon chain in *D. pseudoobscura *(Dp), *D. melanogaster *(Dm) and *A. gambiae *(Ag). Coding exons are represented by red boxes and untranslated UTRs by blue boxes. Introns are not drawn to scale. Because no sufficient information is available about the transcribed non coding sequences of *D. pseudoobscura*, only the coding exons of the *D. pseudoobscura *genes are shown. *mtacp1 *exons duplicated in tandem are labelled 'a' and 'b'. **(c) **alignment of the amino-acid sequences encoded by the duplicate a and b exons of the *mtacp1 *gene in *D. melanogaster *(Dm), *D. pseudoobscura *(Dp), *A. gambiae *(Ag) and *A. mellifera *(Am). Residues conserved in both exons are shown in white on a black background. **(d) **Dendrogram showing the phylogenetic relationships between the duplicated exon DNA sequences used for the alignment shown in (c). The neighbor-joining tree derived from distance matrix analysis was constructed using MultAlin [62]. Other tree-construction methods produced similar results. PAM, percent point accepted mutations.

With the only exception of *Dpse*\*CG5037*, putatively encoding protoheme IX farnesyltransferase, whose 5' genomic sequence was impossible to find in the relevant contig assembly, all other investigated *D. pseudoobscura *genes show a structural organization almost identical to that of their *D. melanogaster *counterparts. Of the 78 *Anopheles *genes studied, 39 maintain the structural organization observed in *Drosophila*, whereas gain or loss of introns occurred in 33, and in six the location of introns is not preserved at all. In agreement with a previous report [4], the intron-exon structure of the gene appears to be conserved in all three dipteran species when splicing of alternative coding exons occurs: the alternative splice forms of both the *Drosophila *NADH-ubiquinone oxidoreductase acyl carrier protein (*mtacp1*, *CG9160*) [27] and the *Drosophila *ATP synthase epsilon chain (*sun*, *CG9032*) [19] have very similar counterparts in *Anopheles*, as shown by genomic structure comparison, alignment of splice variants and EST mapping (Figure 2).

Genes encoding the acyl carrier protein (*mtacp1*) in the three species are characterized by the mutually exclusive use of homologous exons that are repeated in tandem (Figure 2a). The duplicate exons occur at the same location in the aligned amino-acid sequences, and are flanked on both sides by a phase 1 intron. When the sequences of the duplicated exons are compared, they show the expected divergence pattern (that is, the similarity between duplicate exons within a gene is less than the similarity of each exon to its equivalent in the orthologous gene). Evidence from genomic and transcribed sequences (GenBank accession numbers BI510891 and BI508135) shows that the duplicated *mtacp1*exons are also preserved in the more distantly related insect *Apis mellifera *(honeybee) (Figure 2c,d), indicating a specific adaptive benefit for this gene structure, as also suggested by the evolutionary convergence leading to the occurrence of alternative splicing in members of three different ion-channel gene families from *Drosophila *to humans [28]. However, there is no evidence from ESTs that duplicated *mtacp1 *exons undergo alternative splicing in vertebrates and nematodes.

### Analysis of intron length

Interspecies comparison of the introns of putative orthologous genes indicates that there is little constraint on their nucleotide sequence, which undergoes nucleotide substitutions at a rate comparable to that of pseudogenes [29]. However, several observations suggest that intron size is subject to natural selection. For example, in *D. melanogaster *and several other organisms the distribution of intron length has been shown to be asymmetrical, with a large group of introns falling into a narrow distribution around a 'minimal' length and the remaining showing a much broader length distribution, ranging from hundreds to thousands of base-pairs [30-32].

**Figure 3:**
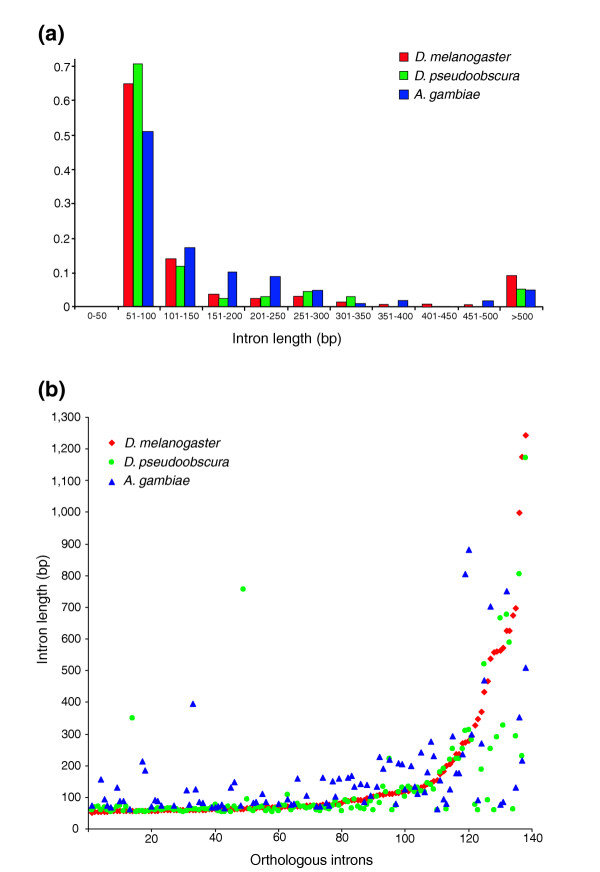
Length distribution of OXPHOS gene introns. **(a) **Length distribution of the 400 introns interrupting the coding sequence in the 78 *D. melanogaster*, *D. pseudoobscura *and *A. gambiae *OXPHOS genes investigated. **(b) **Comparison of the orthologous introns in the three species. Length of 138 *D. melanogaster *introns plotted in ascending length order was compared with the length of the 138 *D. pseudoobscura *orthologous introns and with the length of 98 orthologous *A. gambiae *introns. Note that length class shifts are rare.

Of the introns that interrupt the coding sequence in the 78 OXPHOS genes investigated in the present study, 88 (64.7%) of 136 in *D. melanogaster*, 96 (70.5%) of 136 in *D. pseudoobscura *and 87 (67.9%) of 128 in *A. gambiae *fall into the short-size class (Figure 3a). However, in *A. gambiae *the length distribution of these introns appears slightly broader (62-150 bp, compared with 51-100 bp in both *Drosophila *species). The remaining introns show a broad length distribution, ranging from 151 to 4,702 bp with no clear boundary between classes.

A comparison of the length of introns in corresponding positions in the putative *D. melanogaster*, *D. pseudoobscura *and *A. gambiae *orthologs suggests that changes from the short-size to the long-size (more than 300 bp) intron class, or the converse, have been rare in the evolutionary history of these species: only seven class changes were observed comparing *D. melanogaster *and *D. pseudoobscura *introns, and six between *D. melanogaster *and *A. gambiae *(Figure 3b). On the whole, our data confirm the highly asymmetrical intron length distribution in *D. melanogaster *and extend this finding to the introns of the *D. pseudoobscura *and *A. gambiae *OXPHOS genes.

### OXPHOS gene duplications

It is generally accepted that gene duplication is the basic process that underlies the diversification of genes and the origination of novel gene functions [33]; however, many features of this process are still elusive. To obtain more information on the molecular evolution of the genes involved in the OXPHOS system, we searched the genomes of *D. melanogaster*, *D. pseudoobscura *and *A. gambiae *for duplications of the 78 OXPHOS genes whose orthologs we have identified in the three species.

Duplicate gene pairs were tentatively identified within each genome as best reciprocal hits with an E-value of less than 10^-20 ^in both directions in a TBLASTN search using the default parameters. Deciding whether two proteins may be considered homologous becomes difficult when their sequence identity is within the 20-30% range (the so-called 'twilight zone' [34]), and so the following additional criteria were used: first, the two sequences could be aligned over more than 60% of their length; second, the putative processed proteins encoded had to have more than 40% identity; and third, amino-acid percentage similarity had to be larger than percentage identity [35]. Even if meeting these criteria and reported as different genes in the ENSEMBL database [36], identical *Anopheles *nucleotide sequences were excluded from further analysis, as they are likely to reflect annotation artifacts.

Duplications, or in some instances triplications, of 24 OXPHOS genes were found. Overall, we identified 47 genes (20 in *D. melanogaster*, 19 in *D. pseudoobscura *and eight in *A. gambiae*) each of which shows significant similarity with one of the 78 OXPHOS genes reported above. When the structure of a member of a paralogous gene set indicates that it has been produced by retroposition, it seems reasonable to assume that it is derived from a pre-existing 'parent' gene. For duplicates not clearly originating by retroposition, we also assume, on the basis of the much higher level of conservation and expression, that the genes we find to be the structural orthologs in all three species are the parent ones, and in this case also we will henceforth refer to their paralogs as OXPHOS gene duplicates. The amino-acid percentage identity between the products of duplicate gene pairs ranges from 40% to 85%. For each of the OXPHOS gene duplicates, cytological localization, number of exons interrupting the coding sequence, and number of ESTs found in the *D. melanogaster *and *A. gambiae *EST databases are reported in Table 2. Neighbor-joining trees derived from distance matrix analysis and showing the inferred evolutionary relationship between members of each gene cluster are available at the MitoComp website [22].

Duplications (or triplications) of 16 of the 78 OXPHOS genes investigated were found in both *D. melanogaster *and *D. pseudoobscura*. In such cases, to assign pairwise orthology, besides taking into account conservation of structural organization, given the general conservation of microsyntenic gene order in the two species, we used the products of *D. melanogaster *genes flanking the duplicate loci to search for homologous sequences also flanking the same genes in the *D. pseudoobscura *genome.

The genomic organization of many OXPHOS duplicates shows that they were originated by retropositional events, because they are intronless, or have only very few introns that are likely to have been inserted into the coding sequence after the duplication event. In other cases, duplication apparently resulted from transposition of genomic DNA sequences or from recombinational events, as duplicate genes maintain an identical or very similar structural organization.

On the basis of the presence of the duplication in both species, supported by evidence from evolutionary trees and conservation of microsyntenic gene order, it can be inferred that 15 of the duplications identified occurred before the *D. melanogaster*/*D. pseudoobscura *divergence (about 46 million years ago). On the other hand, five duplications were found only in *D. melanogaster *and four only in *D. pseudoobscura*; in these instances, if the duplication occurred before the divergence of the two species, it has been followed by loss of one of the copies in the lineage leading to the species in which the gene is no longer duplicated. On the assumption that the rate of gene duplication is constant over time, this translates to approximately 0.0014 duplications per gene per million years (4 or 5 duplications per 78 genes per 46 million years) that achieved fixation and long-term preservation in the genome. This value is about twofold lower than the 0.0023 value calculated by Lynch and Conery [37] for the 13,601 genes of the whole genome of *D. melanogaster*. However, it can be argued that the rate of long-term preservation in the genome of OXPHOS gene duplicates cannot be meaningfully compared with the general rate of preservation of duplicates in the whole genome since, while recent data suggest that in eukaryotic genomes there is preferential duplication of conserved proteins [38], duplicates of genes that encode subunits of multiprotein complexes, as most of the genes we have investigated do, negatively influence the fitness of an organism [39], and are therefore unlikely to become fixed in the population. In summary, it appears reasonable to assume that the preservation in the genome of OXPHOS gene duplicates should occur very infrequently, unless special mechanisms allowing their fixation in the population are present (see the next section).

In *A. gambiae *we found only four duplications and two triplications of the OXPHOS genes analyzed; of these, four involve genes also duplicated in one or both *Drosophila *species (Table 2). Pairwise orthology could not be assigned between *Drosophila *and *Anopheles *gene duplicates as neither microsynteny nor evolutionary trees provide sufficient evidence for the origin of the gene pairs from a single-copy gene before the *Drosophila*/*Anopheles *divergence.

### Expression pattern of OXPHOS gene duplicates

The relative abundance of ESTs in a EST library may be assumed roughly to reflect the level of expression of each mRNA in the tissues from which the library was prepared. We therefore used the mRNA sequences predicted *in silico *to be transcribed from the OXPHOS duplicate genes investigated in this work as queries in a search of the public *D. melanogaster *and *A. gambiae *EST databases to infer the relative abundance of the mRNA copies from the hits scored. For each gene, the number of ESTs found in the databases is detailed in Table 2. With the exception of one of the paralogs of the *A. gambiae *gene encoding ubiquinol-cytochrome *c *reductase core protein 1, in all cases the search found the number of ESTs originating from the duplicate gene was strikingly lower than that originating from the putative parent gene, in both *D. melanogaster *and *A. gambiae *(in total, 100 versus 1,747 in *D. melanogaster *and 60 versus 687 in *A. gambiae*). A smaller number of ESTs originating from the OXPHOS gene duplicates was observed even in *A. gambiae *EST libraries that are normalized. Remarkably, and regardless of the mechanism of the duplication, in *D. melanogaster*, in which several organ-specific or developmental stage specific libraries are available, the search showed that the expression of the OXPHOS gene duplicates is strongly testis-biased, as 97 out of the 100 ESTs originating from them were found in testis-derived libraries, while only 27 out of the 1,769 ESTs originating from the parent genes were found in such libraries, the bulk of them being instead found in libraries derived from embryos or somatic tissues.

Our finding that the expression of the OXPHOS gene originated by duplication is strongly testis-biased is validated by the data obtained by Parisi *et al*. [40] using the FlyGEM microarray to identify *D. melanogaster *genes showing ovary-, testis- or soma-biased expression. With the exception of *CG7349*, *CG30354*, *CG30093 *and *CG12810*, for which no data were presented by Parisi *et al*. [40], all other genes reported in this work as OXPHOS gene duplicates were found in the genomic fraction showing testis-biased expression, whereas all the parent genes present in the dataset showed soma-biased expression. Additional data file 2 summarizes the relevant data extracted from Parisi *et al*. [40].

The pattern of strongly testis-biased expression of OXPHOS gene duplicates holds for a further sample of 40 duplications of genes annotated in the MitoDrome database [19] as encoding products that are mitochondrion-targeted but not involved in the OXPHOS system. For 15 of these no data are provided by Parisi *et al*. [40], but all the remaining 25 genes show a testes-biased expression (data not shown).

Duplications of genes encoding OXPHOS subunits, for which stoichiometry is important, are likely to be strongly deleterious owing to the negative consequences of an imbalance in the concentration of the respiratory complex constituents, unless, as proposed by Lynch and Force [41], 'subfunctionalization' and/or a differential expression pattern of duplicate copies occurs. In this case, the duplicate OXPHOS genes would have a reduced or absent capacity to functionally complement mutations in their parent genes, in contrast to what is generally assumed to be the main short-term advantage of gene duplication. In *D. melanogaster *at least there is evidence for this, as FlyBase [42] and BDGP P-Element Gene Disruption Project [43] searches for P-insertion mutants in the *D. melanogaster *OXPHOS genes found that lethal alleles for 11 out of 19 *D. melanogaster *parent genes are known (see the MitoComp website [22]), indicating that loss-of-function of the parent gene cannot be compensated for by the presence of the gene duplicate. P-insertion mutants with an abnormal phenotype, indicating a functional divergence, are known for only one of the *D. melanogaster *OXPHOS gene duplicates - *Cyt-c-d*, encoding cytochrome *c*). Interestingly, although *Cyt-c-d *is adjacent to its putative parent gene, *Cyt-c-p*, it shows a different pattern of expression, suggesting that the two genes must be regulated at individual gene level and not at chromatin domain level (see Table 2).

A systematic investigation of the expression pattern of other *D. melanogaster *duplicate genes will be necessary to answer the question of whether the testis-biased expression pattern reported here is specific to the duplicates of genes encoding mitochondrial proteins, or is a more general phenomenon. According to the balance hypothesis, validated by experimental results obtained on yeast [39], single gene duplications involving genes encoding components of multiprotein complexes are expected to severely affect fitness. Therefore, the expression pattern we have observed could be a necessary condition to maintain some gene duplicates in the *D. melanogaster *genome, at least until they evolve a new useful function. Finally, as nothing is known about the tissue-specific pattern of expression of the genes investigated in *D. pseudoobscura *and *Anopheles*, it also remains unclear whether the testis-biased expression of gene copies originated by duplication is specific to *D. melanogaster*, or is also to be found in other dipterans, and possibly in other organisms.

### Codon usage in the OXPHOS genes

Because of the preferential use of codons ending in C or G, the *D. melanogaster *coding sequences have an average GC content higher than the genomic average [44,45]. This is also true for the 78 *D. melanogaster *OXPHOS coding sequences reported in this work and for their *D. pseudoobscura *and *A. gambiae *counterparts (68% of the codons in the OXPHOS genes end in C or G in *D. pseudoobscura *and 77% in *A. gambiae*, compared to 74% in *D. melanogaster*). In all three species, the coding sequences of OXPHOS gene duplicates show a lower percentage of codons ending in C or G, when compared to both the entire set of 78 orthologous OXPHOS genes and the gene subset including only their parent genes. In samples including all the OXPHOS gene duplicates annotated in this paper the aggregate percentage of C- or G-ending codons is 63%, 46% and 73% in *D. melanogaster*, *D. pseudobscura *and *A. gambiae *respectively, as compared with 70%, 64% and 88% in their parent genes. In *D. pseudoobscura*, the shift toward a higher percentage of A- or T-ending codons is also detected in the pattern of synonymous codon usage; for 12 of the 18 amino acids that are encoded by more than one codon, the most frequently used codon in the *D. pseudoobscura *gene duplicates is different from the one used in their parent genes (see Additional data file 3).

### Chromosomal arm location, interarm homology and microsynteny

It has been reported that in many eukaryotes including yeast [46], *C. elegans *[47], *D. melanogaster *[48,49] and humans [50], genes with related functions and similar expression patterns tend to be clustered, suggesting that they share aspects of transcriptional regulation depending on their inclusion in the same chromatin domain. In particular, Boutanaev *et al*. [48] reported that in *D. melanogaster *clusters of three or more testis-specific genes are much more frequent than expected by chance. Therefore, we investigated the chromosomal distribution of the OXPHOS genes to determine whether clustering could be detected. In all three dipteran species considered, the 78 OXPHOS orthologous genes are randomly distributed on all chromosomal arms (Table 1). Two *D. melanogaster *genes (*Ucrh*, encoding the 11 kDa subunit of ubiquinol-cytochrome *c *reductase, and *CG40002*, encoding the AGGG subunit of NADH-ubiquinone oxidoreductase) have a heterochromatic location.

No evidence of OXPHOS gene duplicate clustering was found either, despite the common testis-biased expression of such genes. Moreover, no evidence of clustering with other testis-specific genes was found when an EST database search for such genes was performed in the regions flanking the investigated gene duplicates.

However, in accord with two studies reporting a significant deficit of genes with a male-biased expression on the *D. melanogaster *X chromosome [51,52], only one out of the 20 *D. melanogaster *OXPHOS gene duplicates, two out of 19 in *D. pseudoobscura *and none (out of eight) in *A. gambiae *were found to be X-linked (Table 2). It may be that duplications of X-linked genes encoding OXPHOS subunits would be especially deleterious because of the male X chromosome transcriptional hyperactivity, which allows dosage compensation.

In all three dipteran species, a disproportionately high fraction of OXPHOS gene duplicates appears to be constituted of autosomal genes derived from parent genes located on the X chromosome (Table 2). As suggested by recent work on the generation and preservation of functional genes produced by retroposition both in *Drosophila *[53] and in the human and mouse genomes [54], this may be explained by a selective advantage for duplicates of X-linked genes that move to an autosomal location and so escape the X inactivation in early spermatogenesis that occurs both in *Drosophila *[55] and in mammals [56].

We would like to speculate that such selective advantage may be especially significant for duplicates of OXPHOS genes, given the heavy reliance of sperm on mitochondrial function. In fact, the excess of autosomal duplicates of X-linked genes is not observed for MitoDrome annotated genes not involved in the OXPHOS system (see above). However, as the general pattern of much lower, testis-biased expression holds even for OXPHOS and other mitochondrial gene duplicates that apparently derive from autosomal parental genes, and even for X-linked duplicates, this pattern (and the explanation of the evolutionary preservation of such genes) cannot only be due to the selective advantage of escaping X inactivation during spermatogenesis.

With the exception of *CG9603*, all euchromatic *D. melanogaster *orthologs maintain their localization on the homologuos *D. pseudoobscura *chromosomal arm (Table 3). *CG9603*, encoding the VIIa polypeptide of cytochrome *c *oxidase, is located on the 3R chromosomal arm in *D. melanogaster*, whereas *Dpse*\*CG9603*, its counterpart in *D. pseudoobscura*, is located on XR; microsyntenic gene order with the flanking genes is conserved in both species, suggesting that a chromosomal rearrangement occurred after their divergence.

OXPHOS gene duplicates also almost always maintain the same chromosomal location and microsyntenic gene order in *D. melanogaster *and in *D. pseudoobscura*. However, a more complex situation was observed with regard to the gene encoding subunit IV of cytochrome *c *oxidase, which is duplicated in *D. melanogaster *and triplicated in *D. pseudoobscura *(Table 2). On the basis of identical genomic organization, conserved chromosomal location and mycrosyntenic gene order *Dpse*\*CG10664 *is inferred to be the ortholog of *D. melanogaster CG10664*. *Dm CG10396*, *Dpse*\*CG10396.1 *and *Dpse*\*CG10396.2 *are intronless, and neither interarm homology nor microsyntenic order offer any clue to their phylogenetic relationship. The dendrogram based on sequence divergence (see the MitoComp website [22], complex IV, subunit IV) suggests, however, that a duplication event occurred before the *D. melanogaster/D. pseudobscura *speciation, originating the *CG10664*-*CG10396 *gene pair (*Dpse*\CG10664-*Dpse*\CG10396 in *D. pseudoobscura*). A further duplication event, occurring in the *D. pseudoobscura *lineage after the *D. melanogaster/D. pseudoobscura *divergence, probably created the *Dpse*\*CG10396.1*-*Dpse*\*CG10396.2 *gene pair.

In contrast to the maintained location of almost all investigated genes on homologous chromosomal arms in the two *Drosophila *species, when *D. melanogaster *and *A. gambiae *are compared the only meaningful correspondence found concerns the genes on the *D. melanogaster *2L and the *A. gambiae *3R chromosomal arms (Table 3). This result is consistent with previous reports that compared the location of homologous genes in *D. melanogaster *and *A. gambiae*, concluding that extensive reshuffling both within and between chromosomal regions has occurred since the divergence of the two species [4,17].

## Conclusions

We have catalogued 78 nuclear genes that control oxidative phosphorylation in three dipteran species and compiled a web-based dataset, MitoComp [22], that contains all the data on which this article is based and which is available with the online version of this article. We have conducted only some basic comparative analyses of the many which are possible using such a dataset, and it is our hope that it will provide a valuable resource for those looking for information about nuclear genes encoding mitochondrion-targeted products in the context of functional genomics and proteomics. Future studies based on this information, especially if the comparative analysis is extended to other species, will surely allow a better understanding of the evolutionary history of a set of genes that control a basic biological function, and also offer interesting insights into the mechanisms of their coordinated expression. In fact, a first *in silico *analysis of the *D. melanogaster *and *D. pseudoobscura *nuclear energy gene sequences suggests that a genetic regulatory circuit, based on a single regulatory element, coordinates the expression of the whole set of energy-producing genes in *Drosophila *[57].

The comparative analysis of the 78 OXPHOS genes in the three dipteran species shows a high level of amino-acid sequence identity, as well as a substantial conservation of intron-exon structure, indicating that these genes are under strong selective constraints. An unexpected and intriguing result of this study is that in *D. melanogaster*, duplication-originated OXPHOS genes are expressed at a much lower level (or possibly not expressed at all) in most or all the tissues where their parent genes are expressed, as judged by the abundance of ESTs derived from their transcripts in all libraries other than those derived from testis. On the other hand, OXPHOS gene duplicates have a strongly testis-biased pattern of expression, a finding validated by other authors with a different approach based on the use of microarrays [40]. In *A. gambiae*, although no testis-specific ESTs databases are available, a pattern of expression of almost all duplicate OXPHOS genes different from that of the gene from which they originated, and possibly limited to specific tissues, is suggested by the fact that in all EST libraries available the abundance of the sequences originated from the duplicate genes is very low when compared with that of the sequences derived from their respective parent genes.

We suggest that, at least in *D. melanogaster*, the acquisition of a new, testis-biased pattern of expression may be required to maintain duplicates of certain genes in the genome. This may also allow rapid acquisition of new functions by the gene product(s), as it has recently been shown that proteins encoded by duplicated genes with a changed expression pattern often show accelerated evolution [58,59]. Subfunctionalization could then further favor the preservation of multiple paralogous genes.

No data are at present available to support the possibility that our findings could be extrapolated to other gene sets or even to the whole genome. However, we propose that duplication of the genes encoding products that are part of multiprotein complexes may be especially deleterious, unless sequence divergence allowing only testis-specific expression of one of the duplicate copies occurs. In turn, this could facilitate the development of novel functions, which is usually assumed to be the main evolutionary advantage of gene duplication, providing a general mechanism for originating phenotypic changes that might also lead to species differentiation.

## Materials and methods

To identify orthologous OXPHOS genes and their duplications in *D. pseudoobscura *and *A. gambiae*, contigs from BCM [13] and scaffolds from AnoBase [21] were searched using TBLASTN with the *D. melanogaster *OXPHOS peptides listed in the MitoDrome database [19] as queries.

Amino-acid sequence identity and similarity values were obtained from pairwise alignments using the Needleman-Wunsch global alignment algorithm at the EMBL-EBI server [60]. Multiple sequence alignments of the OXPHOS amino-acid and coding sequences and visualization of the dendrograms were obtained using the MultAlin 5.4.1 software [61] from MultAlin server [62].

The genomic sequence of each gene was manually searched for intron-exon boundaries and the predicted mRNA sequence reconstructed *in silico*. *A. gambiae *mRNAs were assembled by overlapping ESTs extracted from AnoBase [21].

We have named each newly identified *A. gambiae *gene with the four-letter code 'agEG' followed by the last four or five digits of its Ensembl [36] gene number, excluding the multiple zeros of the prefix; the *D. pseudoobscura *genes were named with the code 'Dpse\CG' followed by the Celera number of their *D. melanogaster *counterparts.

The *D. pseudoobscura *OXPHOS genes investigated here were assigned a chromosomal location where possible, using the putative chromosomal assignments available at BCM [13] for the majority of the large *D. pseudoobscura *contigs. We also utilized the Ensembl mosquito genome server [36] to identify and visualize the chromosomal location of the *A. gambiae *annotated OXPHOS DNA sequences.

The *D. melanogaster *EST database, available from the National Center for Biotechnology Information (NCBI) contains ESTs from cDNA libraries obtained from different developmental stages and body parts. The relative abundance of the transcripts of duplicate or triplicate *D. melanogaster *OXPHOS genes was defined by counting their cognate ESTs in non-normalized cDNA libraries generated by the Berkeley Drosophila Genome Project (BDGP) [43] from embryos (LD), larvae/pupae (LP), and adult ovary (GM), head (GH) and testes (AT), and also the ESTs from adult testes generated at the National Institute of Diabetes and Digestive and Kidney Diseases (NIDDK) [63]. ESTs from BDGP normalized EST libraries generated from head (RH) and embryos (RE) were also considered. The relative abundance of the transcripts of duplicate or triplicate *A. gambiae *OXPHOS genes was defined by counting their cognate ESTs in all libraries recovered from the Anobase server [21]. Since the number of sequences in the EST databases changes as new EST sequences are added, our values are calculated on the EST sequences present in the databases as of July 2004.

The list of *D. melanogaster *P-insertion OXPHOS mutants is reported in the MitoComp website [22] and was mostly compiled using information from FlyBase [42] and from the BDGP P-Element Gene Disruption Project [43].

## Additional data files

A web-based dataset, MitoComp, contains all data on which this work is based and is available at [22]. It includes information on the cytological location of each gene, its genomic organization and the structure of its transcript(s). The genomic structures of the *D. melanogaster*, *D. pseudoobscura *and *A. gambiae *putative OXPHOS orthologs are shown and compared, and their deduced amino-acid products are aligned with the corresponding human protein. When paralogs of the gene exist, neighbor-joining trees derived from distance matrix analysis are also shown to visualize the evolutionary relationships between them. Additional data files available with the online version of this article are as follows. Additional data file 1 contains a table that reports pairwise amino-acid sequence conservation values between the *D. melanogaster *OXPHOS genes investigated and their *D. pseudoobscura*, *A. gambiae *and human counterparts. Additional data file 2 contains data extracted from the Parisi *et al*. dataset [40]. Additional data file 3 reports the codon usage in the orthologous and duplicate OXPHOS genes of *D. melanogaster*, *D. pseudoobscura *and *A. gambiae*.

## Supplementary Material

Additional data file 1 A table that reports pairwise amino-acid sequence conservation values between the *D. melanogaster *OXPHOS genes investigated and their *D. pseudoobscura*, *A. gambiae *and human counterparts Click here for additional data file

Additional data file 2 Data extracted from the Parisi *et al*. dataset Click here for additional data file

Additional data file 3 The codon usage in the orthologous and duplicate OXPHOS genes of *D. melanogaster*, *D. pseudoobscura *and *A. gambiae* Click here for additional data file

## Figures and Tables

**Table 1:** Number of exons and chromosomal localization of the 78 orthologous *D. melanogaster*, *D pseudoobscura *and *A. gambiae *OXPHOS genes

Cluster ID*	Protein name	*D. melanogaster *gene name	Number of exons^†^	Map position	FlyBase ID	*D. pseudoobscura *gene name	Number of exons^†^	Map position	*A. gambiae *gene name	Number of exons^†^	Map position
Complex I: NADH:ubiquinone oxidoreductase											
NUMM	13 kDa A subunit	CG8680	3	2L;25C6	FBgn0031684	Dpse\CG8680	3	4	agEG14117	3	3R;33B
NUFM	13 kDa B subunit	CG6463	3	3L;67E7	FBgn0036100	Dpse\CG6463	3	XR	agEG15380	3	2L22E
NIPM	15 kDa subunit	CG11455	2	2L;21B1-2	FBgn0031228	Dpse\CG11455	2	4	agEG13302	2	3R;35C-D
NUYM	18 kDa subunit	CG12203	3	X;18C7	FBgn0031021	Dpse\CG12203	3	XL	agEG18985	4	2L;27A
NUPM	19 kDa subunit	CG3683	4	2R;60D13	FBgn0035046	Dpse\CG3683	4	3	agEG19249	3	2L;26B
NUKM	20 kDa subunit	CG9172	1	X; 14A5	FBgn0030718	Dpse\CG9172	1	ND	agEG16939	1	X;4A
NUIM	23 kDa subunit	ND23	3	3R;89A5	FBgn0017567	Dpse\CG3944	3	2	agEG9698	2	2R;9A
NUHM	24 kDa subunit	CG5703	3	X; 16B10	FBgn0030853	Dpse\CG5703	3	XL	agEG16953	5	2R;11A
NUGM	30 kDa subunit	CG12079	3	3L;63B7	FBgn0035404	Dpse\CG12079	3	XR	agEG11610	3	2L;24D
NUEM	39 kDa subunit	CG6020	4	3L;77C6	FBgn0037001	Dpse\CG6020	4	XR	agEG18760	3	3L;40A
NUDM	42 kDa subunit	ND42	2	3R;94A1	FBgn0019957	Dpse\CG6343	2	2	agEG10090	2	3L;41C
NUCM	49 kDa subunit	CG1970	6	4;102C2	FBgn0039909	Dpse\CG1970	6	ND	agEG18856	1	X;1B
NUBM	51 kDa subunit	CG9140	4	2L;26B6-7	FBgn0031771	Dpse\CG9140	4	4	agEG9927	4	3R;36D
NUAM	75 kDa subunit	ND75	5	X;7E1	FBgn0017566	Dpse\CG2286	5	XL	agEG19681	4	2R;8D
NI8M	B8 subunit	CG15434	3	2L;24F3	FBgn0040705	Dpse\CG15434	3	4	agEG16251	3	2R;15B
NB2M	B12 subunit	CG10320	2	2R;57F6	FBgn0034645	Dpse\CG10320	2	3	agEG9277	1	3L;46D
NB4M	B14 subunit	CG7712	3	2R;47C6	FBgn0033570	Dpse\CG7712	3	3	agEG12033	2	2R;15A
N4AM	B14.5A subunit	CG3621	2	X; 2D6-E1	FBgn0025839	Dpse\CG3621	2	XL	agEG14707	4	2R;17A
N4BM	B14.5B subunit	CG12400	3	2L:23D3	FBgn0031505	Dpse\CG12400	3	4	agEG16232	3	2R;13C
NB5M	B15 subunit	CG12859	2	2R;51C2	FBgn0033961	Dpse\CG12859	2	3	agEG17759	2	3L;44C
NB6M	B16.6 subunit	CG3446	2	X;5F2	FBgn0029868	Dpse\CG3446	2	XL	agEG7829	3	3R;35A
NB7M	B17 subunit	*l(2)35Di*	3	2L;35D	FBgn0001989	Dpse\CG13240	3	4	agEG18567	3	3R;34D
N7BM	B17.2 subunit	CG3214	4	2L;23A1	FBgn0031436	Dpse\CG3214	4	4	agEG10758	4	3R;31A
NB8M	B18 subunit	CG5548	1	X;13A8	FBgn0030605	Dpse\CG5548	1	XL	agEG8436	3	2L;28C
NI2M	B22 subunit	CG9306	3	2L;34B8	FBgn0032511	Dpse\CG9306	3	4	agEG12344	3	3R;35C-D
ACPM	Acyl carrier	*mtacp1*	4	3L;61F6	FBgn0011361	Dpse\CG9190	4	XR	agEG11237	5	3L;38B
NIAM	ASHI subunit	CG3192	3	X;6C5	FBgn0029888	Dpse\CG3192	3	XL	agEG8821	3	2R;10A
NUML	MLRQ subunit	CG32230	3	3L;80E2	FBgn0052230	Dpse\CG32230	3	XR	agEG12063	3	2R;15A
NINM	MNLL subunit	CG18624	1	X;7C	FBgn0029971	Dpse\CG18624	1	XL	agEG22692	1	X;5A
NIDM	PDSW subunit	*Pdsw*	3	2L;23F3	FBgn0021967	Dpse\CG8844	3	4	agEG7887	4	3R;29A
NISM	SGDH subunit	*l(3)neo18*	4	3L;68F5	FBgn0011455	Dpse\CG9762	4	XR	agEG13573	2	2L;27D
NIGM	AGGG subunit	CG40002	3	ND	FBgn0058002	Dpse\CG40002	3	XR	agEG18653		2R;12D
											
Complex II: Succinate dehydrogenase											
DHSA	Flavoprotein subunit	Scs-fp	4	2R;56D3	FBgn0017539	Dpse\CG17246	4	3	agEG7754	3	3L;38B
DHSB	Iron-sulfur protein	*SdhB*	3	2R;42D3-4	FBgn0014028	Dpse\CG3283	3	3	agEG13539	4	2L;27D
C560	Cytochrome B560 subunit	CG6666	2	3R;86D7-8	FBgn0037873	Dpse\CG6666	2	2	agEG14929	2	3L;39B
DHSD	Cytochrome b small subunit	CG10219	4	3R;95B1	Fbgn0039112	Dpse\CG10219	4	XR	agEG16772	3	X;1C
											
Complex III: Ubiquinol-cytochrome *c *reductase											
UCRY	6.4 kDa protein	CG14482	2	2R;54C9	FBgn0034245	Dpse\CG14482	2	3	agEG12505	2	3L;43B
UCRX	7.2 kDa protein	*ox*	2	2R;49C2	FBgn0011227	Dpse\CG8764	2	3	agEG15210	2	2L;20C
UCRH	11 kDa protein	*Ucrh*	2	3R	FBgn0066066	Dpse\Ucrh	2	2	agEG19398	2	2R;11B
UCR6	14 kDa protein	CG3560	3	X;14B10	FBgn0030733	Dpse\CG3560	3	XL	agEG11611	3	3L;46A
UCRI	Iron-sulfur subunit	*RFeSP*	3	2L;22A3	FBgn0021906	Dpse\CG7361	3	4	agEG16975	4	3R;32C
CY1	Cytochrome c1, heme protein	CG4769	6	3L;64C13	FBgn0035600	Dpse\CG4769	6	XR	agEG19223	4	2L;26C
UCR1	Core protein 1	CG3731	6	3R;88D6	FBgn0038271	Dpse\CG3731	6	2	agEG21302	3	X;5C
UCR2	Core protein 2	CG4169	4	3L;73A10	FBgn0036642	Dpse\CG4169_1	4	XR	agEG17930	4	2L;24A
UCRQ	Ubiquinone-binding protein QP^-^	CG7580	2	3L;74C3	FBgn0036728	Dpse\CG7580	2	XR	agEG20223	2	3L;38C
											
Complex IV: Cytochrome *c *oxidase											
CX41	Polypeptide IV	CG10664	2	2L;38A8	FBgn0032833	Dpse\CG10664	2	4	agEG13327	2	3R;31C
COXA	Polypeptide Va	CoVa	1	3R;86F9	FBgn0019624	Dpse\CG14724	1	2	agEG19581	1	3L;41D
COXB	Polypeptide Vb	CG11015	3	2L;26E3	FBgn0031830	Dpse\CG11015	3	4	agEG8633	4	3R;31C
COXD	Polypeptide VIa	CG17280	2	2R;59E3	FBgn0034877	Dpse\CG17280	2	3	agEG7821	2	X;5A
COXG	Polypeptide VIb	CG18809	1	X;18E5	FBgn0042132	Dpse\CG18809	1	XL	agEG11043	1	2L;25A
COXH	Polypeptide VIc	*cype*	2	2L;25D6	FBgn0015031	Dpse\CG14028	2	4	EST357342	2	3R;29A
COXK	Polypeptide VIIa	CG9603	2	3R;84F13	FBgn0040529	Dpse\CG9603	2	XR	agEG17423	3	X;4B
COXO	Polypeptide VIIc	CG2249	2	2R;46D8-9	FBgn0040773	Dpse\CG2249	2	3	agEG22887	2	2L;28C
											
Complex V: ATP synthase											
ATPA	Alpha chain	*blw*	4	2R;59B1-2	FBgn0011211	Dpse\CG3612	4	3	agEG7500	4	2L;21E
ATPB	Beta chain	ATPsyn-beta	3	4;102D1	FBgn0010217	Dpse\CG11154	3	ND	agEG14379	1	3L;45C
ATPG	Gamma chain	ATPsyn-gamma	1	3R;99B10	FBgn0020235	Dpse\CG7610	1	2	agEG7678	2	3R;29C
ATPD	Delta chain	CG2968	3	X;9B4	FBgn0030184	Dpse\CG2968	3	ND	agEG16076	1	3R;29B
ATPE	Epsilon chain	*sun*	4	X;13F12	FBgn0014391	Dpse\CG9032	4	ND	agEG10095	4	X;3D
ATPF	B chain	ATPsyn-b	3	3L;67C5	FBgn0019644	Dpse\CG8189	3	XR	agEG9580	3	2R;7A
ATPQ	D chain	ATPsyn-d	1	3R;91F	FBgn0016120	Dpse\CG6030	1	ND	agEG10180	3	3L;41C
ATPJ	E chain	CG3321	1	3R;88B4	FBgn0038224	Dpse\CG3321	1	2	agEG10809	3	2L;26B
ATPK	F chain	CG4692	2	2R;60D8-9	FBgn0035032	Dpse\CG4692	2	3	agEG1544	1	ND
ATPN	G chain	*l(2)06225*	2	2L;32C1	FBgn0010612	Dpse\CG6105	2	ND	agEG8590	2	3R;34B
ATPR	Coupling factor 6	ATPsyn-Cf6	2	3R;94E13	FBgn0016119	Dpse\CG4412	2	2	agEG19097	2	2R;19D
AT91	Lipid-binding protein P1	CG1746	3	3R;100B7	FBgn0039830	Dpse\CG1746	3	2	agEG14837	3	X;2B
ATPO	OSCP	Oscp	3	3R;88E8-9	FBgn0016691	Dpse\CG4307	3	2	agEG9393	3	2R;15D
											
Others											
ATPW	ATP synthase coupling factor B	CG10731	1	2R;52F	FBgn0034081	Dpse\CG10731	1	3	agEG15185	1	2R;19B
CI30	Complex I intermediate- associate protein 30	CG7598	2	3R;99B9	FBgn0039689	Dpse\CG7598	2	2	agEG7818	2	X;5A
CYC	Cytochrome C	*Cyt-c-p*	1	2L;36A11	FBgn0000409	Dpse\CG17903	1	4	agEG17602	1	3R;34C
COXZ	Complex IV assembly protein COX11	CG6922	1	2L;25E5	FBgn0031712	Dpse\CG6922	1	4	agEG19985	2	3L;38B
COXS	Complex IV copper chaperone	CG9065	2	X;13A9	FBgn0030610	Dpse\CG9065_1	2	XL	agEG23169	1	3L;44C
OXA1	Biogenesis protein OXA1	CG6404	3	3L;67F1	FBgn0027615	Dpse\CG6404	3	XR	agEG11581	3	2L;22C
ETFA	Electron transfer flavoprotein alpha subunit	*wal*	3	2R;48C1-2	FBgn0010516	Dpse\CG8996	3	3	agEG11798	2	2R;17B
ETFB	Electron transfer flavoprotein beta subunit	CG7834	2	3R;99C1	FBgn0039697	Dpse\CG7834	2	2	agEG13614	2	2R;19D
ETFD	Electron transfer flavoprotein- ubiquinone oxidoreductase	CG12140	5	2R;46C4	FBgn0033465	Dpse\CG12140	5	3	agEG10998	4	2L;23B
COXX	Protoheme IX farnesyltransferase	CG5037	4	2L;31D9	FBgn0032222	Dpse\CG5037	3	ND	agEG11452	4	3R;32B
SCO1	Sco1 protein homolog	CG8885	2	2L;25B5	FBgn0031656	Dpse\CG8885	2	4	agEG10475	1	3R;31C
SUR1	Surfeit locus protein 1	Surf1	4	3L65D4	FBgn0029117	Dpse\CG9943	4	XR	agEG8998	4	2L;25C

*IDs in this column are taken from Swiss-Prot [20]. ^†^Only coding exons were considered. ND, map position not determined. *D. melanogaster*, *D. pseudoobscura *and *A. gambiae *sequences used to determine intron-exon gene structures are available as supplementary material at the MitoComp website [22]

**Table 2:** OXPHOS gene duplications in the genomes of *D. melanogaster*, *D. pseudoobscura *and *A. gambiae*

Protein name	*D. melanogaster *gene name	Number of exons	Number of ESTs*	Map position	*D. pseudoobscura *gene name	Number of exons	Map position	*A. gambiae *gene name	Number of exons	Number of ESTs	Map position
Complex I: NADH:ubiquinone oxidoreductase											
18 kDa subunit	**CG12203**	3		X;18C7	**Dpse\CG12203.1**	3	XL	**agEG18985**	4		2L;27A
					Dpse\CG12203.2	3	2				
20 kDa subunit	**CG9172**	1	36 (1)	X;14A5	**Dpse\CG9172**	1	ND	**agEG16939**	1	47	X;4A
	CG2014	1	0	3R;99B2	Dpse\CG2014	1	2	agEG12298	1	2	2R;14D
24 kDa subunit	**CG5703**	3	33 (2)	X;16B10	**Dpse\CG5703**	3	XL	**agEG16953**	5		2R;11A
	CG6485	1	4 (4)	3L;74A4	Dpse\CG6485	1	XR				
49 kDa subunit	**CG1970**	6	47 (0)	4;102C2	**Dpse\CG1970**	6	ND	**agEG18856**	1	38	X;1B
	CG11913	2	0	3R;96D2	Dpse\CG11913	2	2	agEG19332	1	0	2L;26B
51 kDa subunit	**CG9140**	4	135 (2)	2L;26B6-7	**Dpse\CG9140**	4	4	**agEG9927**	4		3R;36D
	CG11423	1	4 (4)	2R;54C12	Dpse\CG11423	1	3				
	CG8102	2	3 (3)	2R;51F3-4	Dpse\CG8102	2	3				
B14.5A subunit	**CG3621**	2	16 (1)	X;2D6-E1	**Dpse\CG3621**	2	XL	**agEG14707**	4		2R;17A
	CG6914	1	3 (3)	3L;79F2	Dpse\CG6914	1	XR				
											
Complex II: Succinate dehydrogenase											
Flavoprotein subunit	***Scs-fp***	4	54 (0)	2R; 56D3	**Dpse\CG17246**	4	3	**agEG7754**	3		3L:38B
	CG5718	1	5 (14)	3L;68E3	Dpse\CG5718	1	XR				
Iron-sulfur protein	***SdhB***	3	83 (0)	2R;42D3-4	**Dpse\CG3283**	3	3	**agEG13539**	4		2L;27D
	CG7349	3	14 (12)	X;17F3	Dpse\CG7349	1	XL				
											
Complex III: Ubiquinol-cytochrome c reductase											
Cytochrome C1, heme protein	**CG4769**	6	246 (3)	3L;64C13	**Dpse\CG4769**	6	XR	**agEG19223**	4		2L;26C
	CG14508	1	7 (7)	3R;99A1	Dpse\CG14508	1	2				
11 kDa protein	***Ucrh***	2	16 (0)	3R	***Dpse*\*Ucrh***	2	2	**agEG19398**	2		2R;11B
	CG30354	1	1 (1)	2R;44E2							
14 kDa protein	**CG3560**	3	8 (0)	X;14B10	**Dpse\CG3560**	3	XL	**agEG11611**	2		3L;46A
	CG17856	1	0	3R;98C3							
Core protein 1	**CG3731**	6		3R;88D6	**Dpse\CG3731**	6	2	**agEG21302**	3	56	X;5C
								agEG10358	1	2	2R;9A
								gEG15332	1	46	2L;22D
Core protein 2	**CG4169**	4		3L;73A10	**Dpse\CG4169.1**	4	XR	**agEG17930**	4		2L;24A
					Dpse\CG4169.2	1	XR				
											
Complex IV: Cytochrome *c *oxidase											
Subunit IV	**CG10664**	2	138 (3)	2L;38A8	**Dpse\CG10664**	2	4	**agEG13327**	2		3R;31C
	CG10396	1	9 (7)	2R;41F3	Dpse\CG10396.1	1	2				
					Dpse\CG10396.2	1	XL				
Polypeptide VB	**CG11015**	3	41 (0)	2L;26E3	**Dpse\CG11015**	3	4	**agEG8633**	4		3R;31C
	CG11043	2	4 (4)	2L;26E3	Dpse\CG11043	2	4				
Polypeptide VIA	**CG17280**	2	90 (2)	2R;59E3	**Dpse\CG17280**	2	3	**agEG7821**	2	63	X;5A
	CG30093	1	1 (1)	2R;52D3				agEG4851	1	0	3R;32A
Polypeptide VIIA	**CG9603**	2	30 (0)	3R;84F13	**Dpse\CG9603**	2	XR	**agEG17423**	3		X;4B
	CG18193	2	4 (4)	3R;84F13							
											
Complex V: ATP synthase											
Beta chain	**ATPsyn-beta**	3	484 (6)	4;102D1	**Dpse\CG11154**	3	ND	**agEG14379**	1		3L;45C
	CG5389	3	3 (3)	3L;72D5-6	Dpse\CG5389	3	XR				
Epsilon chain	***sun***	4	11 (0)	X;13F12	**Dpse\CG9032**	4	ND	**agEG10095**	4	15	X;3D
	CG12810	1	0	3R;85F11				agEG20782	4	6	3R,34C
								agEG8173	1	0	2L;21D
G chain	***l(2)06225***	2	90 (6)	2L;32C1	**Dpse\CG6105**	2	ND	**agEG8590**	2		3R;34B
	CG7211	2	1 (1)	2L;28C2	Dpse\CG7211	2	4				
Coupling factor 6	**ATPsyn-Cf6**	2	55 (0)	3R;94E13	**Dpse\CG4412**	2	2	**agEG19097**	2		2R;19D
	CG12027	2	2 (2)	3L;64C4	Dpse\CG12027	1	XR				
Lipid-binding protein P1	**CG1746**	3		3R;100B7	**Dpse\CG1746**	3	2	**agEG14837**	3	408	X;2B
								agEG12441	3	4	3L;42A
											
Others											
Complex IV, copper chaperone	**CG9065**	2		X;13A9	**Dpse\CG9065.1**	2	XL	**agEG23169**	1		3L;44C
					Dpse\CG9065.2	1	4				
Cytochrome c	***Cyt-c-p***	1	134 (0)	2L;36A11	**Dpse\CG17903**	1	4	**agEG17602**	1		3R;34C
	*Cyt-*c-d	1	25 (25)	2L;36A11	Dpse\CG13263	1	4				

*The number of ESTs in testis-derived libraries is in parentheses. Because insufficient information on *D. pseudoobscura *ESTs is available in the public EST databases, only *D. melanogaster *and *A. gambiae *ESTs were considered. Bold type is used to identify the putative orthologous genes in the three species (see text). Only coding exons were considered. ND, location not determined. *D. melanogaster*, *D. pseudoobscura *and *A. gambiae *OXPHOS sequences used are available at the MitoComp website [22]

**Table 3:** Chromosomal location and interarm homology of the orthologous *D. melanogaster*, *D. pseudoobscura *and *A. gambiae *OXPHOS genes

	*D. pseudoobscura *chromosomal arm						*A. gambiae *chromosomal arm					
		
	2	3	XL	XR	4	ND	2L	2R	3L	3R	X	ND
*D. mel. *2L 18→					**16**	2		1	1	**16**		
*D. mel. *2R 15→		**15**					6	3	4		1	1
*D. mel. *3L 12→				**12**			7	2	3			
*D. mel. *3R 16→	**15**			1			1	5	4	1	5	
*D. mel. *X 14→			**11**			3	3	4	2	2	3	
*D. mel. *4 2→						2			1		1	
*D. mel. *ND 1→				1				1				

The first column shows the distributions of the OXPHOS genes on *D. melanogaster *chromosomal arms (*D. mel*). Arrows show the direction of counting; *D. melanogaster *→ *D. pseudoobscura *or *D. melanogaster *→ *A. gambiae*. Bold type is used when inter arm homology is conserved betweeen two species. Note that *Dm *2L, *Ag *3R is the only correspondence between *D. melanogaster *and *A. gambiae *chromosomal arms. ND, location not determined.
