# Supplementary material for: Comparison of the oxidative phosphorylation (OXPHOS) nuclear genes in the genomes of Drosophila melanogaster, Drosophila pseudoobscura and Anopheles gambiae
Source: Genome Biol. 2005 Jan 31;6(2):R11. doi: 10.1186/gb-2005-6-2-r11 (PMC551531; doi:10.1186/gb-2005-6-2-r11)
Supplement: Additional data file 3 — The codon usage in the orthologous and duplicate OXPHOS genes of D. melanogaster, D. pseudoobscura and A. gambiae [file gb-2005-6-2-r11-s3.doc]

## Supplemental Table 3. Codon usage of the OXPHOS genes in *D. melanogaster,* *D. pseudoobscura* and *A. gambiae*. For the three Diptera codon usage is tabulated for the 78 OXPHOS genes in this study, and for the duplicate genes and the respective putative “founder” genes. For *D. melanogaster* codon usage is tabulated for all genes in Release 3 [65]. Values are the percent usage of codons per degenerate sense amino acid family; Met and Trp are not included since they have only one codon each. Values in bold indicate the most frequently used codon per amino acid family.

|  |  | Release  3 | OXPHOS  genes  78 | Paralogous genes | | OXPHOS  genes  78 | Paralogous genes | | OXPHOS  genes  78 | Paralogous genes | |
| --- | --- | --- | --- | --- | --- | --- | --- | --- | --- | --- | --- |
|  |  |  |  | Founders 19 | Duplicates 20 |  | Founders 17 | Duplicates 19 |  | Founders 6 | Duplicates 8 |
| Amino Acid | Codon | D. mel. | D. mel. | D. mel. | D. mel. | *D.pse.* | *D.pse.* | *D.pse.* | *A. gam.* | *A. gam.* | *A. gam.* |
|  |  |  |  |  |  |  |  |  |  |  |  |
|  |  |  |  |  |  |  |  |  |  |  |  |
| Ala | GCA | 17.3 | 9.2 | 10.7 | 16.3 | 11.0 | 10.8 | 23.7 | 10.9 | 4.8 | 16.1 |
|  | GCC | **44.5** | **56.5** | **50.4** | **48.8** | **53.7** | **46.7** | 26.6 | **45.6** | **42.0** | **37.1** |
|  | GCG | 18.8 | 14.0 | 13.3 | 15.0 | 10.9 | 11.2 | 21.5 | 29.0 | 39.4 | 36.1 |
|  | GCT | 19.4 | 20.3 | 25.6 | 19.9 | 23.4 | 31.3 | **28.2** | 14.5 | 13.8 | 10.7 |
|  |  |  |  |  |  |  |  |  |  |  |  |
| Arg | AGA | 9.4 | 3.8 | 4.6 | 7.6 | 4.8 | 3.8 | 5.7 | 4.0 | 3.3 | 4.3 |
|  | AGG | 11.7 | 7.9 | 6.1 | 10.3 | 5.7 | 4.1 | 4.3 | 2.5 | 0.0 | 2.1 |
|  | CGA | 15.6 | 8.6 | 9.6 | 12.5 | 6.7 | 7.5 | 5.3 | 8.4 | 5.4 | 7.1 |
|  | CGC | **32.4** | **47.6** | **42.5** | **33.4** | **46.2** | **43.6** | 16.8 | **43.4** | **53.3** | **42.6** |
|  | CGG | 15.0 | 9.3 | 10.4 | 13.7 | 8.5 | 7.2 | **23.5** | 19.1 | 31.5 | 25.5 |
|  | CGT | 16.0 | 22.8 | 26.8 | 22.5 | 28.1 | 33.8 | 14.4 | 22.6 | 6.5 | 18.4 |
|  |  |  |  |  |  |  |  |  |  |  |  |
| Asn | AAC | **54.9** | **71.2** | **69.8** | 49.4 | **64.4** | **64.3** | **51.4** | **78.6** | **84.9** | **73.5** |
|  | AAT | 45.1 | 28.8 | 30.2 | **50.6** | 35.6 | 35.7 | 48.6 | 21.4 | 15.1 | 26.5 |
|  |  |  |  |  |  |  |  |  |  |  |  |
| Asp | GAC | 46.7 | **51.1** | 48.4 | 36.7 | 48.5 | 41.0 | 41.8 | **58.3** | **77.7** | **54.5** |
|  | GAT | **53.3** | 48.9 | **51.6** | **63.3** | **51.5** | **59.0** | **58.2** | 41.7 | 22.3 | 45.5 |
|  |  |  |  |  |  |  |  |  |  |  |  |
| Cys | TGC | **70.4** | **80.7** | **78.3** | **64.2** | **79.7** | **76.2** | **61.3** | **72.6** | **84.0** | **80.0** |
|  | TGT | 29.6 | 19.3 | 21.7 | 35.8 | 20.3 | 23.8 | 38.7 | 27.4 | 16.0 | 20.0 |
|  |  |  |  |  |  |  |  |  |  |  |  |
| Gln | CAA | 30.4 | 20.3 | 23.5 | 28.0 | 23.0 | 28.3 | **85.1** | 15.3 | 7.7 | 28.0 |
|  | CAG | **69.6** | **79.7** | **76.5** | **72.0** | **77.0** | **71.7** | 14.9 | **84.7** | **92.3** | **72.0** |
|  |  |  |  |  |  |  |  |  |  |  |  |
| Glu | GAA | 33.4 | 19.7 | 21.3 | 31.4 | 23.5 | 27.3 | **80.5** | 30.3 | 13.0 | 30.4 |
|  | GAG | **66.6** | **80.3** | **78.7** | **68.6** | **76.5** | **72.7** | 19.5 | **69.7** | **87.0** | **69.6** |
|  |  |  |  |  |  |  |  |  |  |  |  |
| Gly | GGA | 28.6 | 26.8 | 26.4 | **37.4** | 18.5 | 17.2 | **32.8** | 15.1 | 10.3 | 25.0 |
|  | GGC | **42.3** | **47.4** | **42.1** | 29.8 | **51.1** | **51.1** | 29.5 | **47.4** | **59.8** | **36.4** |
|  | GGG | 7.6 | 3.4 | 6.5 | 8.7 | 4.5 | 4.9 | 20.5 | 9.4 | 15.9 | 15.9 |
|  | GGT | 21.5 | 22.4 | 25.0 | 24.1 | 25.9 | 26.8 | 17.2 | 28.1 | 14.0 | 22.7 |
|  |  |  |  |  |  |  |  |  |  |  |  |
| His | CAC | **59.8** | **66.5** | **65.3** | **58.9** | **62.5** | **67.4** | 39.7 | **70.6** | **91.9** | **71.4** |
|  | CAT | 40.2 | 33.5 | 34.7 | 41.1 | 37.5 | 32.6 | **60.3** | 29.4 | 8.1 | 28.6 |
|  |  |  |  |  |  |  |  |  |  |  |  |
| Ile | ATA | 19.5 | 7.7 | 8.2 | 14.2 | 10.3 | 10.7 | 20.5 | 4.4 | 1.4 | 7.1 |
|  | ATC | **46.5** | **62.8** | **56.8** | **47.6** | **48.8** | 42.7 | **41.1** | **70.5** | **88.7** | **61.6** |
|  | ATT | 34.1 | 29.5 | 35.0 | 38.2 | 40.9 | **46.6** | 38.4 | 25.1 | 9.9 | 31.3 |
|  |  |  |  |  |  |  |  |  |  |  |  |
| Leu | CTA | 9.2 | 4.4 | 4.8 | 8.1 | 5.0 | 6.0 | 22.1 | 4.4 | 0.7 | 5.2 |
|  | CTC | 15.3 | 15.9 | 15.6 | 16.1 | 14.7 | 13.8 | 19.7 | 16.8 | 16.9 | 13.4 |
|  | CTG | **42.4** | **53.5** | **52.1** | **42.4** | **47.5** | **40.5** | **25.3** | **63.2** | **76.8** | **60.8** |
|  | CTT | 10.1 | 7.6 | 7.7 | 10.5 | 8.0 | 8.9 | 23.4 | 5.8 | 2.1 | 4.7 |
|  | TTA | 4.9 | 2.7 | 4.0 | 4.0 | 3.9 | 7.0 | 2.2 | 1.2 | 0.7 | 1.9 |
|  | TTG | 18.1 | 15.9 | 15.8 | 18.9 | 20.9 | 23.8 | 7.3 | 8.6 | 2.8 | 14.0 |
|  |  |  |  |  |  |  |  |  |  |  |  |
| Lys | AAA | 29.8 | 14.3 | 13.0 | 25.3 | 21.6 | 20.1 | **73.3** | 18.0 | 8.2 | 23.7 |
|  | AAG | **70.2** | **85.7** | **87.0** | **74.7** | **78.4** | **79.9** | 26.7 | **82.0** | **91.8** | **76.3** |
|  |  |  |  |  |  |  |  |  |  |  |  |
| Phe | TTC | **62.0** | **79.6** | **77.7** | **58.8** | **70.1** | **65.1** | 46.5 | **80.4** | **86.8** | **64.3** |
|  | TTT | 38.0 | 20.4 | 22.3 | 41.2 | 29.9 | 34.9 | **53.5** | 19.6 | 13.2 | 35.7 |
|  |  |  |  |  |  |  |  |  |  |  |  |
| Pro | CCA | 25.3 | 16.5 | 22.2 | 21.2 | 24.0 | 26.7 | 28.7 | 11.9 | 8.4 | 21.6 |
|  | CCC | **32.7** | **50.5** | **41.3** | **32.8** | **47.8** | **41.1** | 17.4 | 18.2 | 8.4 | 12.5 |
|  | CCG | 29.0 | 22.8 | 21.4 | 30.7 | 16.3 | 18.6 | 21.7 | **65.6** | **81.7** | **62.5** |
|  | CCT | 13.0 | 10.2 | 15.1 | 15.3 | 11.9 | 13.6 | **32.2** | 4.3 | 1.5 | 3.4 |
|  |  |  |  |  |  |  |  |  |  |  |  |
| Ser | AGC | **24.5** | 21.3 | 16.6 | 13.6 | 22.1 | 19.9 | 14.9 | 23.3 | 18.2 | 16.7 |
|  | AGT | 14.1 | 7.8 | 5.5 | 12.1 | 6.0 | 5.1 | 9.3 | 5.3 | 0.9 | 8.8 |
|  | TCA | 9.6 | 4.7 | 8.8 | 8.5 | 8.6 | 8.7 | 11.1 | 3.4 | **44.5** | 4.4 |
|  | TCC | 23.4 | **33.2** | **28.6** | **33.4** | **25.8** | **23.7** | **25.4** | 24.5 | 4.5 | 27.2 |
|  | TCG | 20.0 | 25.6 | 28.1 | 20.6 | 24.5 | 25.6 | 15.9 | **39.5** | 26.4 | **41.2** |
|  | TCT | 8.5 | 7.4 | 12.4 | 11.8 | 13.0 | 17.0 | 23.4 | 4.0 | 5.4 | 1.7 |
|  |  |  |  |  |  |  |  |  |  |  |  |
| Thr | ACA | 19.7 | 12.0 | 13.5 | 12.0 | 18.4 | 20.5 | 14.1 | 5.0 | 2.9 | 13.0 |
|  | ACC | **37.4** | **54.4** | **48.2** | **49.0** | **45.4** | **38.6** | **32.1** | 42.1 | 45.7 | 32.0 |
|  | ACG | 25.6 | 17.8 | 17.1 | 21.6 | 19.0 | 16.5 | 26.6 | **45.3** | **47.1** | **47.0** |
|  | ACT | 17.3 | 15.8 | 21.2 | 17.4 | 17.2 | 24.4 | 27.2 | 7.6 | 4.3 | 8.0 |
|  |  |  |  |  |  |  |  |  |  |  |  |
| Tyr | TAC | **62.6** | **78.4** | **81.0** | **64.6** | **65.5** | **62.8** | **63.1** | **84.6** | **98.0** | **74.2** |
|  | TAT | 37.4 | 21.6 | 19.0 | 35.4 | 34.5 | 37.2 | 36.9 | 15.4 | 2.0 | 25.8 |
|  |  |  |  |  |  |  |  |  |  |  |  |
| Val | GTA | 10.9 | 5.4 | 5.6 | 8.5 | 7.7 | 11.3 | 17.5 | 6.4 | 6.2 | 11.4 |
|  | GTC | 23.2 | 29.3 | 26.1 | 18.3 | 28.3 | 26.2 | 28.6 | 32.0 | 38.9 | 17.4 |
|  | GTG | **47.1** | **49.8** | **44.3** | **54.5** | **44.3** | **39.8** | 24.9 | **48.9** | **47.8** | **55.0** |
|  | GTT | 18.8 | 15.5 | 24.0 | 18.7 | 19.7 | 22.7 | **29.0** | 12.7 | 7.1 | 16.2 |
